# Endosymbiotic selective pressure at the origin of eukaryotic cell biology

**DOI:** 10.7554/eLife.81033

**Published:** 2022-11-10

**Authors:** Parth K Raval, Sriram G Garg, Sven B Gould

**Affiliations:** 1 Institute for Molecular Evolution, Heinrich-Heine-University Düsseldorf Dusseldorf Germany; 2 Evolutionary Biochemistry Group, Max-Planck Institute for Terrestrial Microbiology Marburg Germany; IMol Polish Academy of Sciences Poland; Pennsylvania State University United States

**Keywords:** endomembrane system, mitochondria, eukaryogenesis, FECA, LECA, endosymbiosis

## Abstract

The dichotomy that separates prokaryotic from eukaryotic cells runs deep. The transition from pro- to eukaryote evolution is poorly understood due to a lack of reliable intermediate forms and definitions regarding the nature of the first host that could no longer be considered a prokaryote, the first eukaryotic common ancestor, FECA. The last eukaryotic common ancestor, LECA, was a complex cell that united all traits characterising eukaryotic biology including a mitochondrion. The role of the endosymbiotic organelle in this radical transition towards complex life forms is, however, sometimes questioned. In particular the discovery of the asgard archaea has stimulated discussions regarding the pre-endosymbiotic complexity of FECA. Here we review differences and similarities among models that view eukaryotic traits as isolated coincidental events in asgard archaeal evolution or, on the contrary, as a result of and in response to endosymbiosis. Inspecting eukaryotic traits from the perspective of the endosymbiont uncovers that eukaryotic cell biology can be explained as having evolved as a solution to housing a semi-autonomous organelle and why the addition of another endosymbiont, the plastid, added no extra compartments. Mitochondria provided the selective pressures for the origin (and continued maintenance) of eukaryotic cell complexity. Moreover, they also provided the energetic benefit throughout eukaryogenesis for evolving thousands of gene families unique to eukaryotes. Hence, a synthesis of the current data lets us conclude that traits such as the Golgi apparatus, the nucleus, autophagosomes, and meiosis and sex evolved as a response to the selective pressures an endosymbiont imposes.

## Introduction

‘*A scientist in his laboratory is not a mere technician: he is also a child confronting natural phenomena that impress him [her] as though they were fairy tales*’ (Marie Curie).

In evolutionary biology, the transition from prokaryotic to eukaryotic life was a true game changer. Eukaryogenesis involves the origin of new cell biology, genetics, and an unprecedented emergence of morphological diversity. Historically, the prokaryote-eukaryote divide was based on observed differences in morphology and in turn defined this aboriginal branch in the tree of life by their lack of traits that eukaryotes posses (Stanier and Van Niel, 1962). Phylogeny and biochemistry separate prokaryotes into bacteria and archaea (Fox et al., 1980; Koga et al., 1998; Woese et al., 1990) and document the dichotomy of pro- and eukaryotes, which is further evident in the number of protein families (Rebeaud et al., 2021), average protein length (Brocchieri and Karlin, 2005), cellular and morphological complexity (Stanier et al., 1963), and the overall prevalent contribution to the planet’s biomass (Bar et al., 2018).

For decades the field of eukaryogenesis speculated on the existence of a eukaryotic lineage with intermediate biology bridging the prokaryote-eukaryote divide, an elusive grade known as archezoa (Cavalier-Smith, 1987). For curious reasons (see Martin et al., 2017a, for details) this search focused on a eukaryotic phylum lacking a mitochondrion (Cavalier-Smith, 1987; Speijer, 2020), but not necessarily one lacking a nucleus or endoplasmic reticulum (ER). Varying models, but with the common theme of promoting an autogenous origin of a last eukaryotic common ancestor (LECA) independent of a bacterial partner, were proposed (reviewed in Martin et al., 2015). Through the identification of hydrogenosomes and mitosomes (reduced mitochondria; Tovar et al., 1999; Williams et al., 2002) and modern phylogenomics (Burki et al., 2020), we now understand that the biology of LECA matched that of extant garden variety protists. This might appear trivial from todays’ perspective, but reaching this consensus and settling on a mitochondrion-bearing LECA took decades. LECA evolved from an archaeal host cell and its endosymbiotic alphaproteobacterial partner (Imachi et al., 2020; Wu et al., 2022; Zaremba-Niedzwiedzka et al., 2017) and could have been syncytial and fungus-like, with the first gametes budding off as a selectable unit, in what one could describe to be a flagellated protist (Garg and Martin, 2016; Skejo et al., 2021).

The field exploring eukaryogenesis has moved on to studying the nature and origin of the first eukaryotic common ancestor (FECA). This subtle change in terminology has far-reaching consequences. The term FECA only puts a label on the first lineage that we would no longer define as prokaryotic, but which had not yet evolved all traits characterizing the LECA. But at what point did prokaryotic evolution transition into eukaryotic origin? Was it upon the emergence of meiosis and sex? Or the ER and its specialized compartment the nucleus? Or after the transition from archaeal to eukaryotic (bacterial-type) membrane lipids? The transition between prokaryotes and eukaryotes was fluid in nature, with the emergence of the new traits occurring in a currently unknown order (Gould et al., 2016; López-García and Moreira, 2020; Vosseberg et al., 2021). The critical question is: what drove the emergence of eukaryotic traits and what fixed them in evolution?

Here, we discuss the scenarios of a morphologically simple FECA versus a complex one on the basis of reviewing models and data that emerged after the report of the asgard archaeal superphylum from which the eukaryotic host lineage stems. We explore key eukaryotic traits and the phylogenetic distribution of protein associated families, in light of housing an endosymbiont that differs by all other traits in that it represents a semi-autonomous living entity. This imposed unique challenges onto the host throughout eukaryogenesis and whose solution, we argue, is witnessed in the form of compartmentalization, meiosis, and sex.

### FECA and theories of eukaryogenesis in light of the asgard archaea

‘*It can be considered a relatively harmless habit, like eating peanuts, unless it assumes the form of an obsession; then it becomes a vice*’ (Roger Y Stanier).

**Figure 1.**
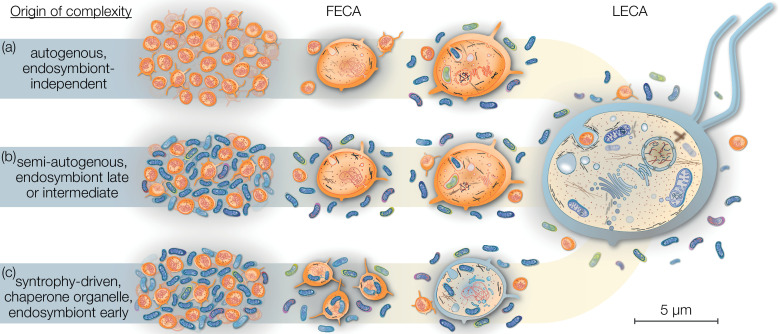
Eukaryogenesis scenarios with respect to the origin and nature of the first eukaryotic common ancestor (FECA). Scenarios summarized in (**a**) were based on the assumption that eukaryotic groups existed that lack mitochondria and which originated independently of endosymbiosis. Such models are no longer supported and we understand that the last eukaryotic common ancestor (LECA) was a mitochondrion-bearing lineage. Models in (**b**) speculate on a (semi-) autogenous origin of complexity. By their definition, the FECA was free of vertically transmittable endosymbionts. Genomic and cellular complexity of FECA grew through horizontally donated genes from diverse prokaryotes part of its habitat; to a degree FECA was eukaryote-like prior to endosymbiosis. Models falling under (**c**) differ from (**b**) in that both partners at the onset of eukaryogenesis were prokaryotic in nature and that complexity only began to evolve after endosymbiosis of the mitochondrial ancestor. By this definition, the FECA is characterized by the endosymbiotic event and describes the lineage that transitioned from prokaryotic to eukaryotic evolution. Note that the prokaryotic cells on the left and the LECA on the right are shown to scale on the basis of average cell diameters.

The relatively harmless habit of tracing the origins of the eukaryotic cell has occupied scientists across several generations, a historical account of which is beyond the scope of this review but has been summarized elsewhere (Martin, 2017). Current models of eukaryogenesis differ above all in the relative placement and contribution of the endosymbiont and consequently the cellular complexity of the host archaeon prior to endosymbiosis. Briefly, mitochondria-early theories place endosymbiosis closer to or at FECA (Figure 1), suggesting that the traits that characterize LECA evolved after endosymbiosis from a prokaryotic-like host cell. On the contrary, mitochondria-late scenarios view endosymbiosis and mitochondrial origin as a finishing touch to the LECA (Figure 1). Intermediate models are gaining popularity, but are often vague on which traits evolved prior or after endosymbiosis.

The notion that eukaryote-like complexity was a prerequisite to phagocytosis for promoting mitochondrial origin appears mandatory to some, but the idea remains unsubstantiated (Leão et al., 2018; Martin et al., 2017b; Mills, 2020; Shiratori et al., 2019). Mitochondria-lacking but phagocytosing LECA models – such as the archezoa hypothesis (Cavalier-Smith, 1987) – lost support due to the now known universal presence of mitochondria across the diversity of all eukaryotic super groups (Hjort et al., 2010; Stairs et al., 2015), but variations of the archezoa hypothesis populate the literature, rekindled on the basis of inferred proteomes from asgard archaea.

Reports of a patchy distribution of homologues of the eukaryotic ESCRT-III, a ubiquitin modifying system, and eukaryote-like actin in the TACK superphylum, triggered thoughts about phagocytosing archaea (Guy and Ettema, 2011; Yutin et al., 2009). The identification of proteins with homology to ESCRT I and II, longin domains, sec23 and sec24 (Zaremba-Niedzwiedzka et al., 2017), Rab-like GTPase (Akıl and Robinson, 2018; Klinger et al., 2016; Surkont and Pereira-Leal, 2016), or profilin that can inhibit (in vitro) rabbit actin polymerization (Akıl and Robinson, 2018; Survery et al., 2021) quickly channelled into speculations that asgard archaea might have a dynamic cytoskeleton, intracellular membrane trafficking, and are morphologically complex (Klinger et al., 2016; Neveu et al., 2020; Zachar et al., 2018; Zaremba-Niedzwiedzka et al., 2017). These interpretations mirror a FECA that is reminiscent of the host lineage at the centre of the archezoa hypothesis (discussed in Martin et al., 2017b), culminating in the depiction of a mitochondrion-lacking eukaryote on the cover of Nature (Pittis and Gabaldón, 2016). When transmission electron microscopy revealed images of asgard archaea, that of *Prometheoarchaeon syntrophicum*, they uncovered tiny prokaryotes with no intracellular eukaryotic traits living in syntrophy with bacteria (Imachi et al., 2020). Such images contradict the narrative of complex asgard archaea, but resonate well with early warnings regarding overinterpretations of metagenome data (Dey et al., 2016).

Eukaryogenesis models rapidly adapted to the discovery of asgard archaea. They now focus on FECA with various speculations regarding the roles of the discovered genes in host biology prior to endosymbiosis. While the level of cellular complexity is not always explicitly declared, several cases can be made out that depict FECA without an endosymbiont (Baum and Baum, 2020; Dacks et al., 2016; Eme et al., 2017; Pittis and Gabaldón, 2016; Vosseberg et al., 2021). Some models can be interpreted one way or the other (Imachi et al., 2020), while some explicitly state that the host cell was a bona fide prokaryote and that eukaryotic traits and biology evolved after endosymbiosis (Gould et al., 2016; López-García and Moreira, 2020; Wu et al., 2022). Notably, the differences among these models rest upon a few dozen genes from the pan-asgard archaeal genome repertoire, whose overall unique contribution to the roots of eukaryotes was 0.3% or less (Knopp et al., 2021; Liu et al., 2021). Sources and timing of gene acquisition in the FECA to LECA transition are equally essential to correctly quantify as they remain hard to predict.

### Entangled branches connecting kingdoms

Among eukaryotic genomes there are more genes of bacterial than of archaeal origin (Alvarez-Ponce et al., 2013; Brueckner and Martin, 2020; Makarova et al., 2005). An autogenous origin of cellular complexity on the basis of an archaeal (host) source alone would predict the opposite but prokaryotes are characterized by mosaic genomes due to horizontal gene transfer (HGT) whose contribution to cellular complexity prior to endosymbiosis is debated (Martin et al., 2017b; Pittis and Gabaldón, 2016). Claims concerning differential loss of genes in extant archaea (Koonin and Yutin, 2014; Eme et al., 2017) are at odds with pangenomes that support a pan-asgard concept (Knopp et al., 2021; Liu et al., 2021). Dynamic genomes and the time passed since eukaryote origin challenge phylogenomic approaches and can skew interpretations including the timing of compartment origin. The estimated timing of gene duplications that depend on molecular clock techniques that are error-prone (Graur and Martin, 2004; Rodríguez-Trelles et al., 2001; Tiley et al., 2020) with respect to the origins of cellular complexity are also debated (Tria et al., 2021; Vosseberg et al., 2021). A reliance purely on relative branch lengths concluded that mitochondrial metabolism and the ER in eukaryogenesis ensued the origin of the nucleus (Pittis and Gabaldón, 2016). The method used has been questioned (Martin et al., 2017a), and the use of unspecific COG (cluster of orthologous genes) annotations in the study is problematic. The few universal proteins listed might operate in the present-day nucleus, but provide little to no evidence for the presence of one prior to endosymbiosis. Proteins of the nuclear pore complex, of which there are about three dozen (Raices and D’Angelo, 2012), were not identified or discussed, nor was the fact that the nucleus is a specialized compartment of the ER from which it forms during cell division (Anderson and Hetzer, 2008). Substitution rates that challenge molecular clock studies vary substantially across species (Baer et al., 2007; Halligan and Keightley, 2009) and the functional unit a protein is associated with (Hartwell et al., 1999). Considering that thousands of new protein families emerged at eukaryote origin that fall into such categories (Preisner et al., 2018) further highlights the caution with which we need to digest molecular clock studies on eukaryogenesis.

**Figure 2.**
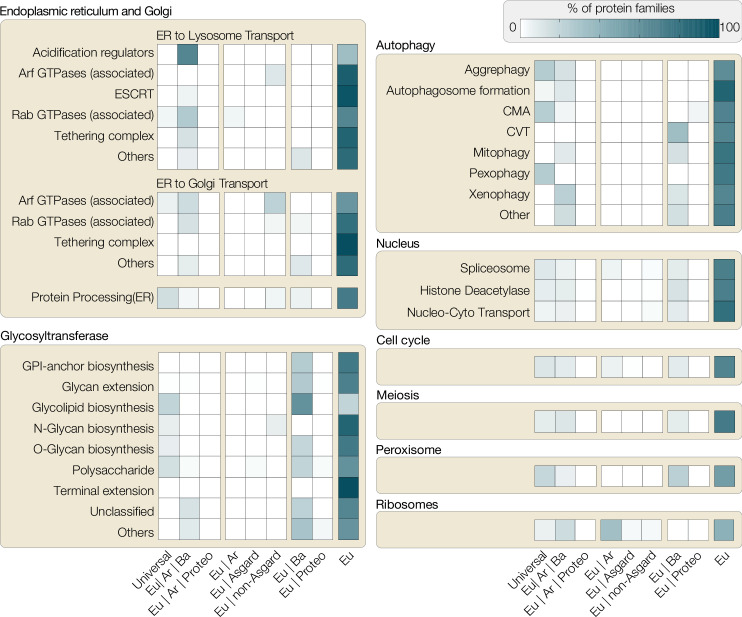
Distribution of protein families involved in eukaryotic traits. All eukaryotic protein sequences associated with different functions in KEGG (Kyoto Encyclopedia of Genes and Genomes, as of November 2021) were used to build representative hidden Markov models (HMMER/3.1), which were then searched against all prokaryotic genomes available on KEGG. Based on hits found across bacterial phyla, the following categories were assigned: Universal (shared with >50% bacterial phyla and archaea), Eu|Ar|Ba (shared with archaea and <50% bacterial phyla), Eu|Ar|Pr (shared with archaea and proteobacteria only), Eu|Ar (shared with Ar), Eu|Asgard (shared only with asgard archaea), Eu|non-Asgard (shared only with non-asgard archaea), Eu|Ba (shared with bacteria), Eu|Proteo (shared only with proteobacteria), Eu (not shared with any prokaryotes). Each box shows the percentage of protein families across these categories (x-axis) for the pathways analysed. For example, a substantial percentage of ribosomal proteins (bottom right) are shared between Eukaryote-Archaea or Eukaryote-Archaea-Bacteria, highlighting the host’s ribosomal contribution to eukaryotes. Proteins for other categories, however, are either predominantly eukaryote-specific or shared between eukaryotes and bacteria (e.g. glycotransferases or some proteins of autophagy). CMA, chaperone-mediated autophagy; CVT, cytoplasm to vacuolar targeting pathway.

The distribution of protein families associated with eukaryotic traits across the domains of life is always telling. Seventy percent or more protein families involved in major eukaryotic traits (such as cell cycle, meiosis, autophagy, nucleus) are specific to eukaryotes, 10–15% (e.g. kinases) are universal across all domains of life, 10–15% are bacterial (e.g. aminopeptidases, mTOR interacting proteins, glycosyltransferases), and a small fraction appear to originate from archaea (DNA licensing proteins of cell cycle, ARG GTPases, *N*-glycan biosynthesis). The distribution of protein families across prokaryotes and eukaryotes (Figure 2) confirms that eukaryotes acquired genes from bacterial or archaeal sources and co-opted them to suit new eukaryotic traits evolving in the FECA to LECA transition, but the majority of protein families involved in eukaryotic cellular complexity are absent across the entire realm of prokaryotic diversity (Brunk and Martin, 2019; Dell et al., 2010; Knopp et al., 2021; Liu et al., 2021; Lombard, 2016). Hence, HGT falls short at explaining the pro- to eukaryote transition with respect to the origin of thousands of eukaryote-unique gene families and a reason for their positive selection in the absence of an endosymbiont. Beyond question, HGT fed into eukaryogenesis – after all, the eukaryotic cell is the product of two prokaryotes – but endosymbiotic partners bring along thousands of genes and many were integrated into the host genome (Timmis et al., 2004). They can explain the pronounced non-alphaproteobacterial signal among proteins supporting eukaryotic traits, especially if we place mitochondrial origin at the root of the FECA and accept HGT to be prevalent.

Phylogenetic trees built using concatenated gene sequences boost phylogenetic signals, but under the premise that the individual genes used recapitulate the evolutionary history of the species (Robinson and Foulds, 1981). For incomplete and contaminated metagenomes (including early releases of asgard archaeal ones), the individual ribosomal gene trees were incongruent (Garg et al., 2021). Similar to simulated chimeric genomes containing genes from different species, metagenome assembled genomes are prone to assembly and binning artefacts. The frequent use of automated pipelines and poorly fitting phylogenetic models exacerbates the risk of drawing false conclusions from metagenome data (Williams and Embley, 2014). For instance, the presence of glycerol-3-phosphate lipids in asgard archaea was claimed (with far-reaching implications on the lipid transition during eukaryogenesis) based on the predicted presence of enzymes involved in the synthesis of ester-linked fatty acids (Villanueva et al., 2017). No evidence of such lipids, however, was found in the biochemical analysis of a cultured asgard archaeon (Imachi et al., 2020) and the presence of the set of required enzymes in asgard archaea has yet to be identified. Better assembly methods result in more complete circular genomes from both axenic culture and metagenomic approaches that mitigate issues of tree congruence (Garg et al., 2021), albeit leaving the same room for interpretations.

Underpinning studies of evolutionary history are phylogenetic trees and theories behind constructing and interpreting them. While it is well beyond the scope of this manuscript to discuss all the vagaries of the field of cladistics and modern phylogenies, it is increasingly evident that many phylogenetic studies have moved from a field that requires expertise in biology to a field that requires expertise in computation (Fitzhugh, 2016) – hypotheses generated from DNA sequences run the risk of taking precedence over morphological evidence (Wheeler et al., 2013). This is less of a critique than a realization. Although sequencing and computational techniques have made significant progress over the years, for the timescales dealt with in early evolution, most issues and challenges remain. It is critical to remember that phylogenetic trees are hypotheses on the evolutionary relationship between organisms and not an observation on itself (Hennig, 2000). No phylogenetic tree is perfect, few are for eternity, and no tree alone will ever satisfy the need for empirical evidence.

### Eukaryotic traits in light of accommodating a prokaryotic endosymbiont

‘*In the case of living machinery, the ‘designer’ is unconscious natural selection, the blind watchmaker*’ (Richard Dawkins).

**Figure 3.**
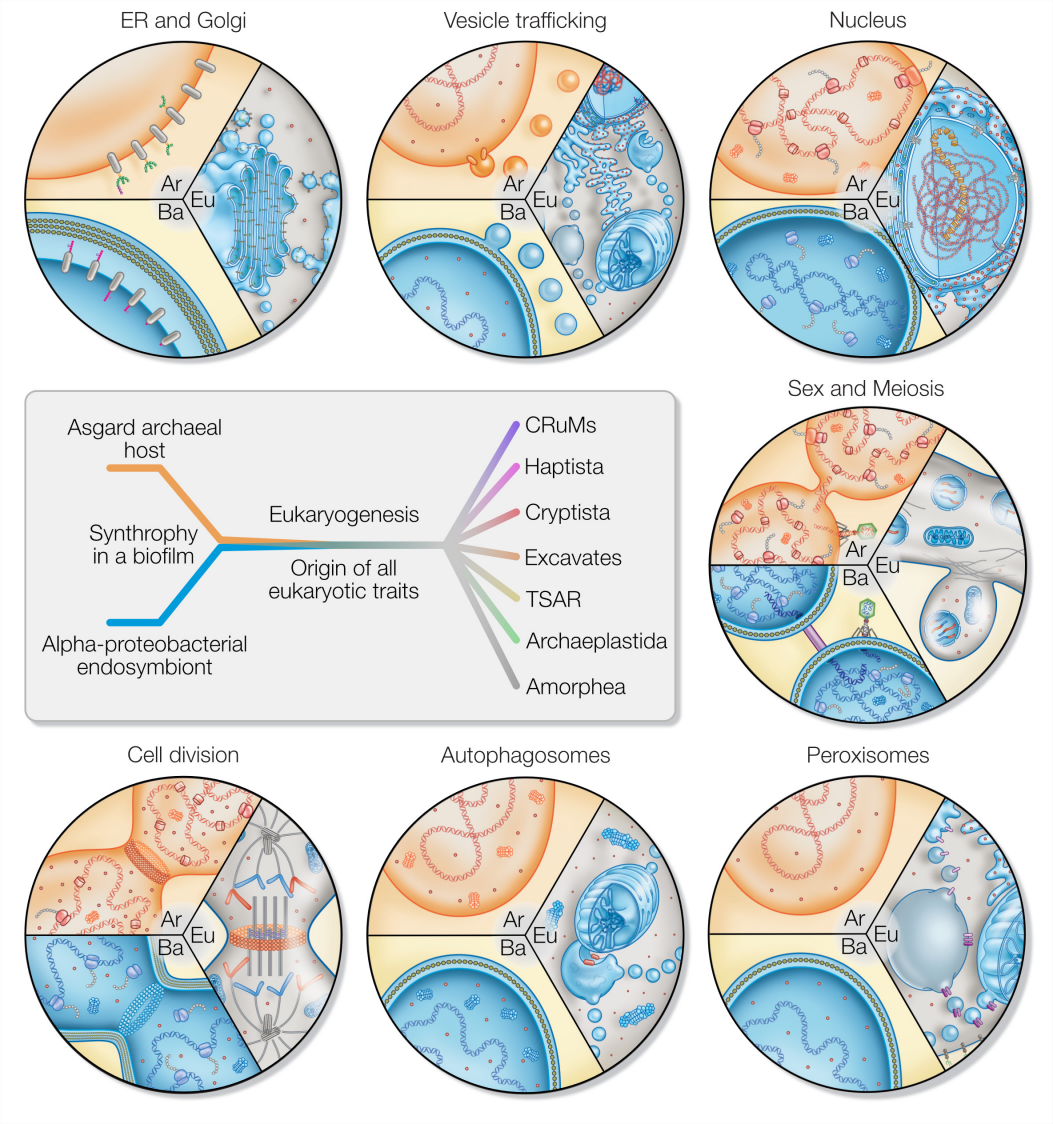
Eukaryotic traits in light of housing an endosymbiont. Each segment highlights an eukaryotic (Eu) trait and the comparable, if present, situation in bacteria (Ba) and archaea (Ar). (**i**) Prokaryotes secrete outer membrane vesicles (OMVs) and an endosymbiont (mitochondrion) secreting OMVs could have (ii) given rise to a dynamic endomembrane system within the archaeal host and explaining the transition from archaeal to bacterial lipids. *N*-glycosylation has been identified in all domains of life, but the eukaryotic *N*-glycosylation pathway is homologous to that of archaea. (iii) A specialized extension of the endoplasmic reticulum (ER), the nucleus prevents co-transcriptional translation of proteins – as is the rule in prokaryotes – to allow for the splicing of introns. (iv) Prokaryotes constantly shed and acquire DNA from the environment, and often promiscuously by transformation, transduction, and conjugation. In the absence of such dedicated mechanisms, eukaryotes avoid Muller’s ratchet through sex and meiosis whose origin might be linked to coordinating the merging of two genomes and synchronizing nuclear and mitochondrial division. (**v**) Peroxisomes also form through mitochondria-derived vesicles and house enzymes of alphaproteobacterial origin. (vi) Eukaryotes perform autophagy using membranes and proteins of the ESCRT machinery to surround and digest internal membrane compartments including the mitochondrion. (vii) While bacteria use homologs of tubulin to perform fission, eukaryotic fission utilizes actin and components of the ESCRT machinery similar to archaea, whereas the tubulin in eukaryotes is for instance used to separate chromatin and intracellular compartments.

Evolution is typically understood to progress gradually and randomly through mutations and the selection of beneficial traits vertically across generations (Darwin and Murray, 1859; Futuyma, 1986). Endosymbiosis adds a massive horizontal component to evolution that is, however, still subject to the basic selection and fixation process. In other words, while the emergence of eukaryotic traits was gradual, the selective pressure that demanded their emergence was more radical. It is this duality that stands between eukaryogenesis theories like a firewall. Any hypothesis that pictures an archaeal lineage transitioning from prokaryotic to eukaryotic cell biology – even of an intermediate type – in the absence of an endosymbiont needs to explain why it was a singularity. Microbial syntrophy is the norm and so is the selective pressure to optimize it. Why are intermediate cell types not observed among the many syntrophic prokaryotes studied, if it was not for the lack of an endosymbiotic event? A mitochondrion-lacking but complex FECA explains eukaryotic traits solely from a host perspective and misses to provide a plausible reason for selection and the emergence and fixation of traits we here discuss in more detail (Figure 3).

#### The ER and Golgi apparatus

Glycosyltransferases are promiscuous enzymes and it has been suggested they are separated through ER-Golgi compartmentalization for that reason (Biswas and Thattai, 2020). *N*- and *O*-glycosylation are ubiquitous in eukaryotic cells, but not so in prokaryotes. Eukaryotic *N*-glycosylation is likely derived from the archaeal ancestor, while *O*-glycosylation is more prevalent among bacteria (Abu-Qarn et al., 2008; Dell et al., 2010; Jarrell et al., 2014). Hence, if each pathway stems from one of the prokaryotic partners, natural selection would foster a spatial separation only upon and not prior to endosymbiosis. The ER lumen and mitochondrial intramembrane space (the former bacterial periplasm) share notable homologies. This includes calcium storage (Dominguez, 2004; Raffaello et al., 2016), disulfide relay systems (Backes et al., 2019), and redox balance (Cardenas-Rodriguez and Tokatlidis, 2017). The contact sites of the ER and mitochondrion are cornerstones for the synthesis and regulation of lipids and a plethora of cellular roles (Booth et al., 2016; Flis and Daum, 2013; Friedman et al., 2011; Hamasaki et al., 2013). This could be a consequence of the ER stemming from mitochondrial-derived vesicles (MDVs) (Gould et al., 2016). MDVs could have provided the necessary endomembrane material for compartmentalization and remain the most plausible source for the lipid transition from ether-linked, archaeal head groups to ester-linked (bacterial) eukaryotic head groups. Much on the origin of the endomembrane system remains a speculation, but not so the existence of MDVs, their role in eukaryotic biology, and how they induce compartment formation (Schuler et al., 2021; Sugiura et al., 2017; Sugiura et al., 2014; Yamashita et al., 2016). Eukaryogenesis models failing to acknowledge their existence miss a biological fact with significant explanatory power.

#### Vesicle trafficking

Vesicle secretion from the plasma membrane into the environment is a common trait of all cells. Unique to eukaryotes are the many ways with which they can internalize membrane vesicles of various sizes, ranging from clathrin-mediated endocytosis (~100 nm) to phagocytosis (>750 nm), using different molecular machineries. Intracellular vesicle trafficking connects the plasma membrane with the endomembrane system and the compartments thereof among each other. All compartments that define the endomembrane system – with the ER at its core – as well as the majority of regulatory and structural proteins are conserved across eukaryotes and absent in prokaryotes (Klinger et al., 2016; Kontou et al., 2022).

#### The nucleus

The nucleus is a distinctive extension of the ER and forms from the latter after mitosis (Anderson and Hetzer, 2008) using homologs of ESRCT complex (Olmos et al., 2015). It separates transcription from translation and is the site of pre-ribosome assembly (Peña et al., 2017). As with any trait, a selective reason for its presence must outweigh the costs for its maintenance; consider, for example, the exchange of mRNA and effectors with the cytoplasm (Nerurkar et al., 2015; Warner, 1999). A plausible selection could have been imposed by the transfer of group II introns from the endosymbiont that drove the origin of eukaryotic splicing and need for separating transcription slowed by the spliceosome from fast translation (Martin and Koonin, 2006). Mitochondria-early scenarios provide both the problem (group II introns that need to be spliced) and the solution (MDVs that might have given rise to the ER) (Gould et al., 2016).

#### Sex and meiosis

The prokaryotic solution to prevent mutational overload through Muller’s ratchet is HGT (Muller, 1964). The nucleus renders the eukaryotic cytoplasm almost sterile of DNA (preventing HGT), wherein it plays a regulatory immune function (Abe et al., 2019; Paludan and Bowie, 2013). The eukaryotic solution was ploidy, multinucleated cells and reciprocal recombination through meiosis (Garg and Martin, 2016). A multinucleated state is readily achieved by decoupling nuclear from cell division, a mechanism commonly observed in prokaryotes wherein the DNA replicates independently of the cell before portioning into daughter cells (Haeusser and Levin, 2008). The syncytial theory for eukaryotic origin (Garg and Martin, 2016) posits that by virtue of multinucleated cells within a singular archaeal host, multiplying bacterial symbionts are free to lose genes via endosymbiotic gene transfer to the multiple copies of the host nucleus/nuclear material in the cytoplasm to explore various configurations under the constant onslaught of group II introns, yet retaining fitness by compensating viable mRNA in-trans within the same shared cytoplasm. The explanatory power of this model is twofold: (i) it explains how homologous recombination – which subsequently evolved to meiosis as we understand it today – was necessary to maintain viable copies of undisrupted genes, while simultaneously maintaining the presence of bacterial transferred genes, and (ii), it explains the monophyly of eukaryotes. As long as the FECA to LECA transition continued, the multitude of host nuclei remained within a single confined cytoplasm until the fittest version was optimized via various rounds of endo-meiosis and homologous recombination. Any origin of cell division and/or cell cycle might have given rise to gamete-like spores that separated off the original syncytium. In cases where a successful combination was released through ESCRT-driven scission (see below), a similar process applies for further optimization. In scenarios in which the budded off cell (gamete) was fitter than the syncytium, it would outcompete the original syncytium or alternatively would be outcompeted when it contained aberrant genomes. In either case, the singularity of LECA is well explained by the syncytial model of the FECA to LECA transition (Garg and Martin, 2016; Skejo et al., 2021).

Meiosis in itself is ancient, ubiquitous, and the central process that imparts an advantage to sex in eukaryotes (Colnaghi et al., 2022; Colnaghi et al., 2020; Malik et al., 2008; Speijer et al., 2015). Several theories place mito-nuclear interactions, heteroplasmy, and mitochondrial ROS as drivers of eukaryotic sex (Colnaghi et al., 2020; Hörandl and Speijer, 2018; Radzvilavicius and Blackstone, 2015). HGT alone was insufficient for LECA to escape Mullers ratchet in the absence of homologous recombination (Colnaghi et al., 2022), when considering expanding genome size and repeat sequence frequency. Everything points to an origin of sex and meiosis necessitated by the presence of mitochondria. Moreover, sex, as a trait, restricts the number of potential mating partners (by 1/number of sex types), and it is hence less surprising that it did not evolve in groups outside of eukaryotes, but had to in the FECA.

#### Peroxisomes

The majority of enzymes of peroxisomal beta-oxidation are of alphaproteobacterial origin (Bolte et al., 2015) and peroxisomes might have evolved to compartmentalize ROS-producing beta-oxidation and protect the mitochondrial genome (Speijer, 2017). De novo biosynthesis of peroxisomes involves MDVs with integrated Pex3 and Pex14 that fuse with ER-derived vesicles containing Pex16 (Sugiura et al., 2017), and the compartment for beta-oxidation appears absent in species lacking respiring mitochondria (Le et al., 2020). Peroxisomes not only make sense in the presence of a mitochondrion, they are also partly a product thereof (Mohanty and McBride, 2013; Sugiura et al., 2017).

#### Autophagy

Cytosolic protein homeostasis in prokaryotes is performed by proteases and proteasomes, which are common to both archaea and bacteria. Defective membrane proteins and membranes are shed by mechanisms similar to bacterial outer membrane vesicle secretion (Schwechheimer and Kuehn, 2015). Eukaryotes utilize membrane-bound compartments in the form of autophagosomes, also for recycling membranes including their proteins (Nakatogawa, 2020). Mitophagy removes damaged mitochondria and is initiated by ER-mitochondrial contact sites (Hamasaki et al., 2013). It is a trait needed in the presence of large intracellular compartments and the occasional yet inevitable breakdown of organelles that require immediate containment (Anding and Baehrecke, 2017).

#### Cell division

The eukaryotic cell cycle is a series of choreographed steps that leads to the correct portioning of genetic material, endomembrane, and organelles to both daughter cells (Harashima et al., 2013). The presence of a nuclear compartment and an endosymbiont are incompatible with binary fission in the absence of orchestrated replication and organelle and compartment division, and cytokinesis. As mentioned in the previous section, in prokaryotes the nuclear material (genome) replicates independently of cell division, which would have facilitated the formation of syncytial populations (Haeusser and Levin, 2008). During this time, however, we speculate that mitochondrial metabolism started playing a more significant role in controlling cell division, given the role of nutrient availability in coordination of cell division. The G1 phase of the eukaryotic cell cycle results in the mitochondria as the master regulator for S/G2 progression (Antico Arciuch et al., 2012; Mitra et al., 2009), suggestive of a deep link between mitochondria and the cell cycle and one that would have been difficult to integrate into a pre-existing one. Eukaryotic cell division employs the use of ESCRT homologs and actin in contrast to bacterial division mechanisms involving FtsZs from which also tubulin evolved (Christ et al., 2016; Goliand et al., 2014; Stoten and Carlton, 2018). This suggests the evolution of an independent pathway for cell division involving ESCRT proteins consistent with their role in archaeal cell division (Tarrason Risa et al., 2020), one that was based on outer membrane vesicle secretion, but this time packaging mitochondria and the nucleus/genetic material, the latter similar to a role of prokaryotic OMVs (Schwechheimer and Kuehn, 2015). Understanding how these pre-existing mechanisms were leveraged in an elaborate checkpoint system of the eukaryotic cell cycle remains to be elucidated.

One might consider the cytoskeleton another eukaryotic trait, but this is more involved. The eukaryotic cytoskeleton rests on three main pillars: (i) actin and associated proteins, (ii) tubulin and associated proteins, and (iii) the utterly diverse intermediate filament (IF) proteins. Components of each pillar, sometimes also in combination, can be found in archaea and bacteria alike (Duggin et al., 2015; Larsen et al., 2007; Preisner et al., 2018; van den Ent et al., 2001; Wickstead and Gull, 2011; Zaremba-Niedzwiedzka et al., 2017). As with many things in eukaryogenesis, it is the intricate combination and universal presence of all three cytoskeletal pillars and the dynamic nature which they are used in eukaryotes that is characteristic. The latter is best demonstrated by the rapid switch in motility between actin-based gliding and tubulin-based flagella-driven swimming in many protists, likely also a feature of the LECA (Fritz-Laylin et al., 2010; Kusdian et al., 2013; Preisner et al., 2018). Basic components were derived from the host cell, such as gelsolin-regulated actin filaments (Akıl et al., 2020) and evolution co-opted such mechanisms en route to LECA. It is conceivable that with expanding cell size, increased intracellular complexity and the need of an orchestrated cell cycle, the selection for a dynamic but simultaneously in parts rigid cytoskeleton increased, which triggered the expansion of the IF protein family required for mechanical support, and the origin of additional accessory proteins and regulatory mechanisms that are absent in prokaryotes.

The identification and subsequent characterizations of asgard archaea have done the following for eukaryogenesis: (i) They underpin the syntrophic origin of eukaryotes involving two prokaryotic partners and (ii) provide support for a 2D tree of life (i.e. two domains of life, bacteria and archaea, evolved from the origin of life and eukaryotes emerged from within archaea after endosymbiosis of an alphaproteobacterial partner). (iii) They provide no evidence for the presence of bacterial-type ester-linked lipids in asgard archaea, (iv) reject a complex archaeal ancestor necessary to explain the patchy distribution of eukaryogenesis-relevant gene families (Wu et al., 2022), and (v) show that the asgard archaeal set of genes before unique to eukaryotes closes the gap to the number of gene families encoded by eukaryotes by only 0.3% (Knopp et al., 2021) or less (Liu et al., 2021). Hence, with respect to explaining the origin of eukaryotic traits and a rationale for their universal presence in eukaryotes, the asgard archaea and their syntrophic bacterial partners support and place us at scenarios that were submitted some 25 years ago (Martin and Müller, 1998; Moreira, 1998; Vellai and Vida, 1999).

Considering syntrophy as a key ecological parameter in eukaryogenesis was an early notion that has stood the test of time (Imachi et al., 2020; López-García and Moreira, 2020; López-García and Moreira, 2019; Sousa et al., 2016; Spang et al., 2019; Wu et al., 2022). Ever since, observations from the field of microbial ecology, genomics, and geology continue to encourage us to picture eukaryogenesis to have occurred within a microbial mat, where multiple species thrive in close proximity and ample syntrophies exchanging substrates such as H_2_/electrons under limited or no oxygen (López-García and Moreira, 2020). Recent advances in geochemistry added new support to the proposal that eukaryogenesis occurred in anoxic niches with a preferred shift towards aerobic metabolism being a secondarily derived state (Mills et al., 2022) and so do the culturing conditions of Prometheoarchaeon (Imachi et al., 2020). Such prokaryotic consortia can source genes through HGT from pangenomes of other bacteria and (asgard) archaea and the virosphere that also contributed to the birth of the eukaryotic genome (Spang et al., 2022; Wu et al., 2022). Evidently, however, most eukaryotic protein families evolved during the FECA to LECA transition and selective pressures due to endosymbiosis were likely key.

To conclude, physiological and phylogenomic studies support a mitochondria-early scenario and so does cell biology (Figure 3). Claiming mitochondria were of little importance in eukaryogenesis contradicts the simultaneous claim that intermediates lacking mitochondria all went extinct – an oxymoron that suggests that the mitochondrial endosymbiont contributed little in the FECA to LECA transition, while its presence was vital for the survival of FECA during eukaryogenesis.

### On being the right size in eukaryogenesis

‘*The most obvious differences between different animals are differences of size, but for some reason the zoologists have paid singularly little attention to them*’ (John BS Haldane).

**Figure 4.**
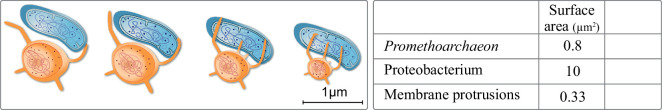
Extracellular membrane protrusions from asgard archaea. Potential trade-off between cytosol investment between cell and protrusion. Each 1 µm increase in protrusion takes 12% cytoplasm, likely limiting total protrusion and reducing cell volume. Values in the table for the asgard archaea were calculated based on Eme et al., 2017: average asgard cell radius: 0.25 µm, average protrusion radius 50 nm, and length 1 µm. Average alphaproteobacterium radius and length were taken to be 0.5 and 3 µm, respectively.

Haldane began his influential essay by addressing a lack of scale bars in zoology books. One can point to a similar issue regarding eukaryogenesis, in which models often depict cells not changing in size up to scale in the course of the FECA to LECA transition (Baum and Baum, 2020; Gould et al., 2016; Spang et al., 2019). Though not intentional, this is important. In eukaryogenesis we are dealing with at least a 10 times increase in cell diameter, with known consequences regarding cell volume, morphology, and molecular diffusion limits among other factors (Young, 2006). Engulfing a proteobacterium with a surface area of 10 µm^2^ requires 10 times the surface area of the asgard archaeon *Prometheoarchaeon*. For a typical protist it is only 1% of its surface area. Putting scales on a recent model, the entangle-engulf-endogenize mode (Imachi et al., 2020) brings forth details worthy considering: an observed tubular protrusion requires 12% of cytoplasm for a 50% increase in surface area interacting with syntrophic partners (Figure 4). Four to six protrusions approximately result in the doubling of cytosolic volume, maybe explaining why *Prometheoarchaeon* has not been observed to produce more than six protrusion per cell.

Such protrusions might have been relevant for the uptake of the symbiont, but the surface area of such a protrusion is 3% that of a proteobacterium. Entangling a proteobacterium entirely would take at minimum 50 protrusions, the cost of which is six times the cytoplasmic volume and not considering the multitude of proteins needed for recognition, surface attachment, and the processes thereof. We also note that we know neither of a case in nature where tubular-like extensions (allowing nutrient exchange) fuse to sheets (allowing cell engulfment), nor can we imagine how this would work on a molecular level, respecting membrane biology, and in 3D. Considering scale bars or tubular versus sheet-like membrane biology is not intended to disprove any model, but it highlights potential issues and also reminds us of the question of when and how did the size increase in the FECA to LECA transition.

### A feed forward loop supporting an increase in cell size

‘*The higher animals are not larger than the lower because they are more complicated. They are more complicated because they are larger*’ (John BS Haldane).

**Figure 5.**
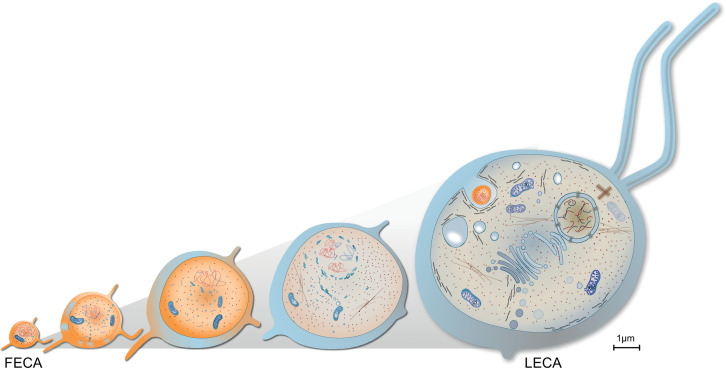
Evolution of last eukaryotic common ancestor (LECA) after the symbiotic event. Vesicles from the proteobacterium accumulated in cytosol giving rise to permeative barrier around the host DNA Brueckner and Martin, 2020, selected positively due to parallel bombardment of genes (Land et al., 2015). As a result, the genome of bacterium shrunk, whereas that of the host expanded, along with increase in cell size. New combinations of genes, powered by energy from the endosymbiont, led to emergence of novel trait and LECA with eukaryotic cell biology.

Haldane noted that an elephant has to be as complicated as an elephant, because it is as large as an elephant. Across eukaryotes, an increase in cell size (1000–10,000 times) and morphological complexity is common, matched by a comparable increase in genome size. The upper end of bacterial genomes is 15 Mb (Land et al., 2015), that of (haploid) eukaryote is around 130 Bb (Pellicer et al., 2010). What drove this increase in cell and genome size during eukaryogenesis (Figure 5)?

In prokaryotes ATP production is limited by the available cell surface. This limits the replication rate and imposes negative selection on genome expansion (Lane, 2007). Conversely, in mitochondria powered eukaryotes, energetic efficiency increases with cell size (Hochachka et al., 2003; Lane, 2007), imposing a positive selection. Increased cell size in eukaryotes means increased DNA content to maintain the karyoplasmic ratio (Cavalier-Smith, 2005; Cavalier-Smith, 1985), which is positively selected for (Lane, 2007). Through this, and remembering that the endosymbiont provided the host cell with both problems and solutions (main text), one can speculate on mechanisms and a selective pressures for the emergence of eukaryotic cell biology, cell and genome size during the FECA to LECA trajectory.

Endosymbiosis provided an influx of endosymbiotic genes and membrane material. An early endomembrane system with minimal protein content, for example, proteins that are likely to be packed for secretion via OMVs, etc., emerged and the nucleus formed for reasons discussed in the main text. Constant fusion of endosymbiont-derived vesicles with the archaeal host provided a mechanism for the lipid shift and compartment origin (Gould et al., 2016), which might have fostered an increase in cell size. Integration of endosymbiotic DNA provided one early mechanism for why the genome size increased, together with duplication events (Kelly, 2021; Tria et al., 2021; Van de Peer et al., 2009). The ongoing concentration of ATP synthesis to mitochondria imposed positive selection on cell size, which allowed for a further increase in genome size. A feed-forward process of increased cell size stipulating increased genome size and vice versa commenced that was supported by an emerging endomembrane system and intracellular transport that counteracted the molecular diffusion limit (Figure 5). It provided ground for new combinations of genes to emerge and expressed (Lane and Martin, 2010) and increased cell size accommodated experimental expression of new proteins in the cytosol (Dill et al., 2011). This presented an opportunity – almost unlimited in theory – for the origin of new protein families and complex traits. Considering that most of these new inventions revolve around the endosymbiont (Figure 3), suggests it drove the selection for their emergence and fixation. Haldane might have put it this way: the LECA got larger because it was complex and it became complex because it was larger.

### Did eukaryogenesis come with a price tag and if so, who paid?

‘*I have not failed. I’ve just found 10,000 ways that won’t work*’ (Thomas Edison).

The cost of innovation is significantly higher than the manufacturing of the final product – the COVID vaccine serves as a topical example (Light and Lexchin, 2021). Eukaryotes have innovated several folds higher number of protein families than archaea as evident from genome (Brueckner and Martin, 2020) and proteome data (Müller et al., 2020) alike, underpinning the complexity across the eukaryotic tree of life. Since mutations lack foresight and are more likely to be deleterious than advantageous (Eyre-Walker and Keightley, 2007), inventing new proteins takes a considerable amount of trial and error. Ribosome production and protein biosynthesis consumes the majority of a cell’s energy budget (Harold, 1986; Kafri et al., 2016) and the energy budget of trial and error would be orders of magnitude higher. Mitochondria were key by providing eukaryogenesis with an energetic freedom that supported this unparalleled level of innovative protein evolution and expression (Lane and Martin, 2010). While challenged (Lynch and Marinov, 2017; Schavemaker and Muñoz-Gómez, 2022), we do not see it disproven (Gerlitz et al., 2018; Lane, 2020). Calculations questioning the bioenergetic contribution of mitochondria do not account for the cost of evolving novel proteins.

The acquisition of a respiratory electron transport chain through excessive HGT does not make a cell complex ( Nelson-Sathi et al., 2012), because the location of the bioenergetic membrane matters. The ratio of bioenergetic membrane (=energy generation) to genome size is high when harbouring an endosymbiont with internalized energetic membranes and a reduced genome (Lane and Martin, 2010). Also, a bioenergetic plasma membrane is incompatible with phagocytosis and the internalization of the bioenergetic membrane was a prerequisite to evolve phagocytosis (Martin et al., 2017b). A physiological observation that puts a timing on events in the FECA to LECA transition. Eukaryotes that maintain complexity in the absence of respiring mitochondria has prompted some to question the importance of mitochondria and a surplus of ATP at eukaryote origin (Hampl et al., 2019), while missing a critical detail: the examples listed stem from species that are either parasites or commensals of eukaryotes and who are energetically dependent on canonical mitochondria. The same holds true for the only eukaryotic taxon not possessing mitochondria, Monocercomonoides. They secondarily lost mitochondria and can only thrive in the gut of some animals (Karnkowska et al., 2016). Such parasites or commensals have engaged an evolutionary path characterized by energetic dependency. Their complexity might diminish over evolutionary timescale, should they not go extinct with their hosts first. The issue is the origin of eukaryote complexity from prokaryotic ancestors, not the maintenance of eukaryotic complexity from eukaryotic ancestors.

An alternative to the energetics argument in explaining the ubiquity of mitochondria and its role in eukaryogenesis is missing, and the papers that question it are no exception. The internalization of energetic membrane – energy production from only 10% of cell volume – decoupled from the genome, as is the case in mitochondria (Fenchel, 2014), provides an optimum for protein innovation and a selection towards a complexity that can maintain a 200 ton blue whale.

### A successful endosymbiosis and origin of a new domain: chance or necessity?

‘*Everything existing in the universe is the fruit of chance and of necessity*’ (Democritus).

Evolution is random and selects for fitness. Extinction is the rule and so is the common principle of use it or lose it (Lahti et al., 2009). Traits that remain unchanged across various organisms and through a billion years of evolution are indicative of the fact they are fundamental. Endosymbiosis is absent among prokaryotes (apart from isolated exceptions) and so is morphological complexity comparable to that of eukaryotes (reviewed in Martin et al., 2017b). Is it by chance or necessity? It is unlikely that the endosymbiosis leading into the origin of the eukaryotic domain was the first and only attempt throughout now 4 billion years of prokaryotic evolution.

The set of challenges posed by an endosymbiont are generic in nature: (i) there is a constant influx of endosymbiotic DNA which, after integration into the host genome (Allen, 2015; Portugez et al., 2018), is also exposed to an increased accumulation of deleterious mutations (Eyre-Walker and Keightley, 2007). The secretion of outer membrane vesicles by the endosymbiont is inevitable (Deatherage and Cookson, 2012), as well as the removal of irreversibly damaged organelles or the need to supply the endosymbiont with substrate from ions to peptides. Dividing endosymbionts need to be integrated into the division cycle of an archaeal host itself relying on simple binary fission.

A lot of solutions are associated with compartmentalization and this is a good time to remember that the mitochondrial endosymbiont not only provided the challenges, but maybe also the material to solve some (Gould et al., 2016). Any attempts at eukaryogenesis are prone to fail, if such challenges are not met by solutions that furthermore require correct timing (Barbrook et al., 2006). An influx of genes via HGT alone does not translate into complexity. Despite the metabolic transformation of haloarchaea via a chunk of some 1000 genes of bacterial origin (Nelson-Sathi et al., 2012) – maybe through a syntrophic partner and failed endosymbiosis – haloarchaea show no intracellular complexity. So while the encounter of the mitochondrial ancestor with an archaeal host occurred by chance, the emergence of a complex cell biology upon endosymbiosis was a necessity. Once a cell biology that can chaperone an endosymbiont is established, however, additional endosymbionts may follow without noticeable changes to the host.

The subsequent acquisition of the plastid added no extra compartments to the heterotrophic host that gave rise to the Archaeplastida, despite adding thousands of cyanobacterial genes to the host genome (Timmis et al., 2004). The same is true for an independent plastid acquisition by a rhizarian protist (Lhee et al., 2019) and likely many other endosymbiont-bearing protists (Husnik et al., 2021). Ever since eukaryogenesis, the cellular framework required for housing another prokaryote was in place. Some compartments have experienced physiological remodelling, such as the peroxisome (Islinger et al., 2010), but many components that evolved to service mitochondria during eukaryogenesis were recycled for the plastid: dynamins for fission (Fujimoto and Tsutsumi, 2014), redox balance through thioredoxins (Thormählen et al., 2017), and organelle digestion and recycling through the autophagosome (Ishida et al., 2014). One could add secondary endosymbioses, in which the acquisition of algae by eukaryotic hosts can lead to the stripping of all eukaryotic compartments of the endosymbionts (including their mitochondria), but that otherwise add no additional compartment or complexity to the host.

### Conclusion

Morphologically complex life on Earth has a singular origin: eukaryogenesis. The LECA had evolved all canonical traits that we understand separates prokaryotic from eukaryotic life. Closing the gap between a simple FECA and a complex LECA by presupposing a complex FECA opens an equally wide gap between a simple and a complex archaeal host. Picturing FECA without an endosymbiont offers little explanation for the existence or emergence of eukaryotic traits and the lack thereof in prokaryotes (including asgard archaea), apart from the inevitable that all eukaryogenesis models face: the need to script the blueprint of a eukaryotic cell. And should then not all (asgard) archaea with a syntrophic partner be considered FECA in the sense that in principle they have the potential to become eukaryotic? For 4 billion years, prokaryotes have overall remained the same in terms of cellular complexity with some rare exceptions having evolved a single compartment type, but nothing vaguely similar to the conserved nature of the eukaryotic endomembrane system. Reflecting on eukaryotic traits and their cell biological connection to mitochondrial origin lets us conclude they are better understood as being selected for to service an endosymbiont and less so as means of acquiring one. Phylogeny guided models should connect interpretations to a physiological and cell biological rationale, while facing the challenge of resolving the fluid nature of the pangenomes of both host and endosymbiont genome throughout eukaryogenesis – we need to talk to phylogenetic trees, not only about them. Physiology (Imachi et al., 2020; Martin and Müller, 1998; Moreira, 1998; Spang et al., 2019), geochemistry (Mills et al., 2022), phylogenetics (Wu et al., 2022), and culturing and imaging (Imachi et al., 2020) all point to a syntrophic origin of eukaryotes involving two prokaryotic partners. The data suggests that first steps towards endosymbiosis in eukaryogenesis were of prokaryotic nature, that eukaryogenesis likely only solidified upon endosymbiosis, and that hence the definition of FECA should include an endosymbiont.

## Figures and Tables

**Appendix 1—figure 1.**
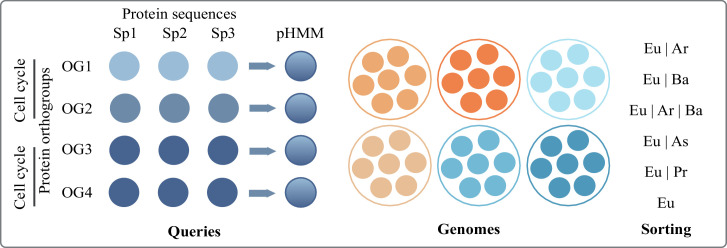
Overview of the pHMM screening pipeline to identify proteins (orthogroups) shared between eukaryotes and varying prokaryotic groups.

## Appendix 1

### Brief methods and criteria for sorting eukaryotic protein families (Figure 2 of the main text)

To screen for protein families involved in eukaryotic complexity in the prokaryotic world, we compartmentalized ‘eukaryotic complexity’ into: ER and Golgi (ER to lysosome transport and ER to Golgi transport), glycotransferases, autophagy, nucleus, cell cycle, meiosis, and peroxisome.

Protein orthogroups (a collection of protein sequences that are likely to perform a close to identical function across species) annotated under each of these categories were taken from the Kyoto Encyclopedia of Genes and Genomes (KEGG). Protein sequences under each of these orthogroups (annotated by unique KEGG Orthology or KO, ID) were obtained from all eukaryotic species available as of November 2021. From multiple protein sequences, hidden Markov models (HMM) were built, each representing a single protein orthogroup. These HMM profiles were used as query against all prokaryotic species available on the database. Searches were conducted using HMMER/3.1.

Bacterial species were categorized into phyla (e.g. Acetobacteria, Cyanobacteria) as per NCBI taxonomy, available also at KEGG. Archaeal species were categorized only into asgard and non-asgard. Each phyla had variable numbers of species and to account for this, we used % of species thresholds instead of number of species threshold. If a match was found in 5% of the species of a given phyla, we called it a match for that phylum to keep our estimates conservative. Protein families that had no hits in >5% species of any bacterial phylum (and asgard and non-asgard archaea) were considered unique to eukaryotes. Protein families present in >50% of bacterial phyla, asgard archaea, and non-asgard archaea were labelled as universal. Between the two extremes of universal and unique to eukaryotes, protein families with hits in a prokaryotic domain were further sorted into categories of relevance (shown in Figure 2, main text) as follows:

**Appendix 1—table 1.** Key to the different groups compared among each other.

Category	Present in
Universal	>50% bacterial phyla, asgard and non-asgard archaea
Eu|Ar|Ba	<50% bacterial phyla, asgard and non-asgard archaea
Eu|Ar|Proteo	Asgard and non-asgard archaea, exclusively proteobacteria (no other bacterial phylum)
Eu|Ar	Asgard and non-asgard archaea, no bacterial phylum
Eu|Asgard	Asgard only (no other archaea or bacteria)
Eu|non-Asgard	Non-asgard only (no other bacteria or asgard archaea)
Eu|Ba	Bacterial phylum other than proteobacteria
Eu|Proteo	Only proteobacterial phyla
Eukaryotes (Eu)	No hits in >5% of any prokaryotic phylum
